# Anderson localization of a one-dimensional quantum walker

**DOI:** 10.1038/s41598-017-18498-1

**Published:** 2018-01-29

**Authors:** Stanislav Derevyanko

**Affiliations:** Department of Electrical and Computer Engineering, Ben Gurion University of the Negev, Beer Sheva, 84105 Israel

## Abstract

We study the evolution of a system performing a one-dimensional quantum walk in the presence of static phase disorder. The same model also describes the propagation of classical light pulses in photonic mesh lattices. We study the interplay between the coupling (i.e. the bias of the “quantum coin”) and disorder. We provide an exact analytical expression for the localization length for two limiting cases of strong and weak phase disorder. In all the cases of interest we supply numerical simulations for participation ratio, Lyapunov exponent and the return probability as functions of the coupling parameter.

## Introduction

The concept of discrete time quantum walk (DTQW) was originally suggested in^1^ and represents a generalization of a classical random walk where instead of “flipping a coin” at each discrete step a quantum walker with at least two internal degrees of freedom undergoes a “quantum coint toss” - a unitary transformation that mixes the components of the wave function. After that the walker moves one position to the “right” or “left” in the configurational space depending on which component was detected. If the system is allowed to evolve freely and no measurement is taken then after *m* discrete time steps the system is in a superposition of multiple “left” and “right” moving states^2–5^. Unlike its deterministic counterpart which is characterised by a diffusive spread of the walker’s position a quantum walker relies on interference and mixing of the left and right components ultimately achieving ballistic spread of the wave function^1^.

After the original advent of the idea it was quickly realised that a DTQW can be most efficiently implemented in optics and does not necessarily require purely quantum settings. In fact it was recognized early on that DTQW is an interference phenomenon that can be realized by purely classical means^6–9^. As the result the discrete time evolution system occurs in many classical spatially and temporally modulated systems like e.g. optical meshes^10–12^ and, of particular interest to our problem, fibre loops^11,13–15^.

The basic properties of the coherent DTQW in mesh lattices and fibre loops are currently well understood and studied both theoretically and experimentally. On the other hand the effects of decoherence and dephasing on the DTQW are less explored area^16–19^. Thus we are interested here in a particular case of *static disorder* implemented as random phase scrambling at each site *n*. This type of disorder was already studied both numerically and experimentally in^15,19,20^ where the onset of Anderson localization (AL)^21^ was reported when the strength of phase fluctuations was large enough. Moreover, rigorous mathematical conditions for the existence of AL in the disordered quantum walks (including very general conditions for the coin probability measure) have been established in works^22,23^. Yet currently there are lacking analytical results for the localization length as the function of system parameters including the coupling strength between the components (i.e the bias of a quantum coin).

In this paper we provide an analytical expression for the Lyapunov exponent (LE) the latter serving a measure of the reciprocal localization length for an arbitrary coupling ratio. In particular for the most popular situation reported in literature, namely, 50:50 power split in optical meshes or loops (corresponding to a “fair” coin) the localization length, *l*_*loc*_, is analytically found to be equal to 2/log  2 = 2.88 which is confirmed by the corresponding numerical simulations. Another analytical result of the current paper is the expression of the Lyapunov exponent in the case of weak phase scrambling of the components of the quantum walker. These results serve as an important criteria for estimating the boundaries of the ballistic regime and its tolerance towards both weak disorder and change of coupling. What sets aside Anderson localization of a quantum walker or a light pulse in a fibre loop from that in the original tight-binding model^21^ or in the coupled optical waveguides^24,25^ is that in the DTQW settings strong additional cross-coupling between the the two components does not inhibit the localization phenomenon but in fact helps achieve better localization. This has serious implication for the finite extent of the walker domain where the localization length can be significantly less than the system size even for very small levels of phase disorder provided that the coupling between the components (loops) is strong enough. This in turn will have an effect on the performance of future devices that might exploit the DTQW phenomena for speed-up of quantum computation^26,27^.

We have also performed numerical simulations of the participation ratio (closely related to the localization length) and the second moment of the field distribution (probability components of a quantum walker) as functions of the number of the evolution steps and quantified the absence of diffusion in the quantum evolution.

## Mathematical model of a quantum walk

Mathematically the evolution of the system during a single step of the DTQW is described by a unitary operator $\hat{{\mathscr{U}}}=\hat{S}\hat{C}$ which consists of two steps. We denote by $|{\rm{\Psi }}\rangle =|n,\alpha \rangle$ the state of the walker which is located at a coordinate position *x* = *n* and having an internal degree of freedom (e.g. spin^1^ or polarization^3^ in the quantum case) *α* = ↑ or ↓ “up” or “down”. In the classical context of optical meshes or fibre loops variable *α* labels upper or lower fibre loop^11,13–15^ or adjacent mesh sites^10–12^. The quantum coin operator $\hat{C}$ is a unitary matrix acting on the spinor components only and is given by the general form

1 $$\hat{C}=(\begin{array}{cc}\cos \,\theta  & {e}^{i\phi }\,\sin \,\theta \\ -{e}^{-i\phi }\,\sin \,\theta  & \cos \,\theta \end{array})$$

Thus the angle *θ* ∈ [0, *π*/2] (or rather sin*θ*) is a measure of cross-coupling between the *U* and *V* components so that *θ* = 0 corresponds to the absence of coupling, while *θ* = *π*/2 is the case of maximum coupling - both being the extreme cases of a biased quantum coin. The position shift operator $\hat{S}$ then shifts the position of the walker to the right or to the left depending on the value of the internal degree of freedom:

2 $$\hat{S}=\sum _{n}\,\{|n+1,\uparrow \rangle \langle n,\uparrow |+|n-1,\downarrow \rangle \langle n,\downarrow |\}$$

We can introduce the “up” and “down” components of the quantum state as *U*_*n*_ = 〈*n*, ↑|Ψ〉, *V*_*n*_ = 〈*n*, ↓|Ψ〉. Introducing the discrete time index *m* we can finally write down the time evolution $|{\rm{\Psi }}(m+\mathrm{1)}\rangle =\hat{U}|{\rm{\Psi }}(m)\rangle$ as a system of coupled equations:

3 $$\begin{array}{rcl}{U}_{n}^{m+1} & = & \cos \,\theta \,{U}_{n-1}^{m}+{e}^{i\phi }\,\sin \,\theta \,{V}_{n-1}^{m}\\ {V}_{n}^{m+1} & = & -{e}^{-i\phi }\,\sin \,\theta \,{U}_{n+1}^{m}+\,\cos \,\theta \,{V}_{n+1}^{m}\end{array}$$

In the popular fibre loop implementation^11,13–15^ the spinor components ${U}_{n}^{m}$ and ${V}_{n}^{m}$ correspond to the classical fields in the two coupled fibre loops of unequal lengths sampled at different time markers *n* after *m* round trips. The common choice is that of the 50:50 power coupler which in the quantum case corresponds to the so called “fair” coin which obtains when *θ* = *π*/4, *φ* = *π*/2. Here however we shall consider the most general case.

The eigenmodes of system (3) can be easily found^10,14^. Looking for a solution in the form of ${({U}_{n}^{m},{V}_{n}^{m})}^{T}={(U,V)}^{T}\,\exp (iQn-i\beta m)$ one obtains the following dispersion law for the propagation constant *β* playing the role of the quasienergy: cos *β* = cos *θ* cos *Q*. One can see that this dispersion law is doubly periodic in both *Q* and *β* with the two bands between *θ* ≤ *β* ≤ *π* − *θ* and *π* + *θ* ≤ *β* ≤ 2*π* − *θ*. The bandgap closes in the absence of cross-coupling when *θ* = 0 and the dispersion law becomes conical which in the limit of continuous time provides the connection with the relativistic (Dirac) evolution^28^.

## Results

### Quantum walk in the presence of phase disorder

One way of implementing static phase disorder is to multiply the coin matrix $\hat{C}$ by an additional diagonal matrix ${\rm{diag}}\,({e}^{i{\varphi }_{\uparrow }(n)},{e}^{i{\varphi }_{\downarrow }(n)})$. The random phases *ϕ*_↑↓_(*n*) in our chosen implementation are independent for different *n* and are sampled from a uniform distribution in the interval [−Φ_*max*_, Φ_*max*_]. The value of Φ_*max*_ or rather the variance of the phase 〈*ϕ*^2^〉 which for the uniform distribution is equal to ${{\rm{\Phi }}}_{max}^{2}/3$ plays the role of the strength of disorder. We shall call the case where Φ_*max*_ = *π* - the case of full (strong) disorder. In optical fibre loops such disorder can be introduced in a controlled way by inserting optical phase modulators in each loop^13,15,19^. In fact as we shall see below in all cases of interest in order to achieve Anderson localization for all the modes it is sufficient to dephase only a single component and put, say *ϕ*_↑_ to zero as was indeed done in^15,19^.

The discrete time evolution (3) is now given by a disordered discrete propagator: $|{\rm{\Psi }}(m)\rangle ={\hat{{\mathscr{U}}}}^{m}|{\rm{\Psi }}\mathrm{(0)}\rangle$ where the unit step operator $\hat{{\mathscr{U}}}$ has been modified to include static phase disorder so that its eigenvectors and eigenvalues are the solutions of the following non-Hermitian (unitary) random eigenvalue problem:

4 $$\begin{array}{rcl}z{U}_{n} & = & {e}^{i{\varphi }_{\uparrow }(n)}(\cos \,\theta \,{U}_{n-1}+{e}^{i\phi }\,\sin \,\theta \,{V}_{n-1})\\ z{V}_{n} & = & {e}^{i{\varphi }_{\downarrow }(n)}(-{e}^{-i\phi }\,\sin \,\theta \,{U}_{n+1}+\,\cos \,\theta \,{V}_{n+1})\end{array},\quad z\equiv {e}^{-i\beta },\quad n=2,\ldots ,\,N-1$$

The above equations should be supplemented with the suitable boundary conditions (BCs) i.e. the equations for the evolution of *U*_1_ and *V*_*N*_. The simplest type of BCs preserving unitarity are of course periodic BC which we will use in most of the direct numerical simulations of systems (3) and (4). For the subsequent Lyapunov exponent analysis a more convenient choice is presented by the free boundary conditions *U*_0_ = *V*_*N*+1_ = 0. In the thermodynamic limit of large *N* both types of BC yield similar dynamics and spectra.

**Figure 1:**
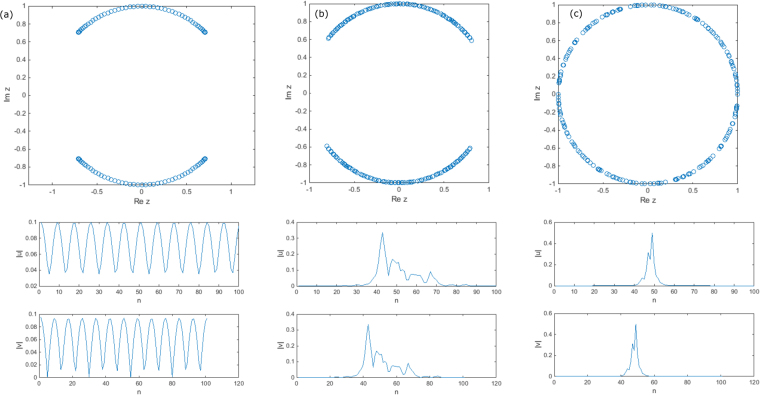
The complex spectrum (first row) and the eigenmode profile (second and third row) of the disordered DTQW for Φ_*max*_ = 0 (**a**), Φ_*max*_ = *π*/7 (**b**), and Φ_*max*_ = *π* (**c**). The values *θ* = *π*/4, *φ* = *π*/2 and *N* = 100 are assumed.

Regardless of the choice of the model for the phases or the boundary conditions system (4) experiences Anderson localization which is particularly easy to observe in the case of full disorder Φ_*max*_ = *π*^15^. This is illustrated in Fig. 1 where we show the eigenspectrum and the eigenmodes of Eq. (4) with periodic boundary conditions, fully uncorrelated phase factors exp(*iϕ*_↑↓_(*n*)) for progressively increasing levels of disorder. The unitarity assures that the eigenvalues are concentrated on a unit circle *z* = exp(−*iβ*) but as one increases the strength of disorder Φ_*max*_ the bandgap closes and the eigenfunctions become localized. One can also observe that the density of states (phase angles *β*) becomes progressively uniform over the whole interval [−*π*, *π*] with the increase of disorder. In each column one relization of disorder and one typical mode is shown. Similar results have been reported earlier in^15^.

Before we continue we shall note a few important symmetries of the stationary eigenvalue problem (4). One such symmetry which was initially discovered numerically is that regardless of the disorder model all the eigenmodes (*U*_*n*_, *V*_*n*_)^*T*^ posses staggered symmetry: |*V*_*n*_| = |*U*_*n*+1_| (apart perhaps from the boundary sites *n* = 1, *N*). This is clearly seen from Fig. 1. Indeed if we plug an ansatz *V*_*n*_ = *U*_*n*+1_ exp(*iφ*_*n*_) into Eq. (4) the compatibility condition between the two equations provides us with the recurrence relation between exp(*iφ*_*n*+1_) and exp(*iφ*_*n*_) which does preserve the unitarity as long as *z* lies on a unit circle (which of course includes the spectrum). One obvious consequence of that is that one only needs to study the localization properties of one component, say *U*_*n*_, since the properties for *V*_*n*_ will be exactly the same.

Let us stress that the strength of disorder Φ_*max*_ is not the only parameter in the system that dictates the localization properties of the eigenmodes. The second parameter is the coupling strength given by the angle *θ*. It is clear from Eq. (4) that when *θ* = 0 i.e. the cross-coupling is absent the localization is also absent and all the states are extended so that |*U*_*n*+1_| = |*U*_*n*_| for each *n* for arbitrary levels of disorder. And in the opposite limit of strong cross-coupling *θ* → *π*/2 all states are always localized with the eigenmodes (*U*_*n*_, *V*_*n*_)^*T*^ proportional to $({\delta }_{n,{n}_{0}},{\delta }_{n,{n}_{0}-1})$ even in the absence of disorder. It is clear then that there is a competition between coupling and disorder so that in the case of strong cross-coupling and strong disorder all states are localized while in the opposite limit the states are extended. It is interesting therefore to study the behavior of the system in the intermediate points in the space of parameters (*θ*,Φ_*max*_). We performed a series of numerical simulations of the localization properties in the space of these parameters. Generally the localization length *l*_*loc*_ is defined via the rate of exponential decay of an eigensate at *n* → ∞. This quantity however is often difficult to measure directly and a much more widely used numerical measure is the participation number *P*_*N*_ defined as^29^:

5 $${P}_{N}=\frac{{({\sum }_{n}{|{U}_{n}|}^{2})}^{2}}{{\sum }_{n}{|{U}_{n}|}^{4}}.$$

Its physical meaning is the volume where a given mode differs significantly from zero. Its maximal value ~*N* corresponds to fully delocalized state while the minimum value of 1 is attained for perfect localization ${U}_{n}={\delta }_{n{n}_{0}}$. The participation number generally represents an upper bound for the localization length: ${P}_{N}\mathop{ > }\limits_{ \tilde {}}{l}_{loc}$ which is due to the fact that some exponentially decaying states can still have an internal structure at the intermediate scale *P*_*N*_ which can be larger than *l*_*loc*_^7^. In fact the evidence of this is clearly seen in Fig. 1(b). Nevertheless the proximity of the *P*_*N*_ to its maximum value *N* is a strong indication of the weakness of the localization and values close to 1 signify the opposite limit of strong localization.

**Figure 2:**
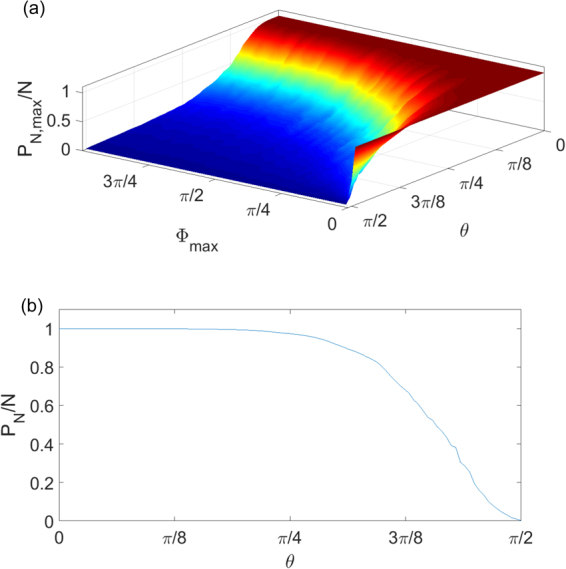
The maximum normalized participation number *P*_*N*_/*N* as a function of the coupling angle *θ* and maximum disorder level Φ_*max*_ (**a**) and its 1D slice (**b**) corresponding to the weak disorder level Φ_*max*_ = 0.1.

In Fig. 2 we plot the results of the simulations of a system consisting of *N* = 500 sites averaged over 10 realizations of disorder to smooth out the residual fluctuations. At each point in the parameter space we have taken the maximum value of the *P*_*N*_ over all eigenmodes of system (4) corresponding to the most extended state. The first observation one makes from Fig. 2(a) is that the *P*_*N*_ grows dramatically as the cross-coupling becomes weaker, *θ* → 0, especially for small levels of disorder. To magnify this effect in Fig. 2(b) we present a 1D slice of the same graph for small values of disorder. One can see that starting from the values of the coupling angle *θ* ≈ 1.1 and up to the boundary value *π*/2 the participation number drops dramatically which signifies the onset of localization. This boundary is also confirmed by the theoretical result for the weak-disorder Lyapunov exponent (see Eq. (11)) below.

We defer the study of the dynamical properties of the DTQW and the measure of the spread of the initially localized wavepackets until further sections and consider next a convenient representation for the localized eigenmodes of system (4).

### The tridiagonal representation

In the following part of the paper we build a theory of the localization of disordered quantum walk based on the three-diagonal representation of the stationary quantum walker problem.

As we have seen in the previous section, unless the angle *θ* is close to zero system (4) shows strong coupling between the components *U* and *V*. We can however obtain an autonomous system of equations for each component alone (save for the boundary values). Since we have shown that it is sufficient to study the localization of one component only one can substitute e.g, *V*_*n*−1_ from the second equation Eq. (4) into the first one and then use the first equation again to eliminate *V*_*n*_ altogether. Of course similar manipulations can be performed to obtain an autonomous system for *V*_*n*_ only.

Note that since we are mostly interested in the localization phenomenon i.e. the exponential decay of tails of the eigenmodes it is the intensity distribution of the eigenmodes that matters. Therefore we can introduce a transformation ${\tilde{U}}_{n}={U}_{n}\,\,\exp (-i{\varphi }_{\uparrow }(n))$ and for the transformed quantity one has an equation:

6 $$\cos \,\theta \,{e}^{i{\varphi }_{\downarrow }(n-\mathrm{1)}}\,{\tilde{U}}_{n+1}-{z}^{-1}{e}^{i{\varphi }_{\downarrow }(n-\mathrm{1)}+i{\varphi }_{\uparrow }(n)}{\tilde{U}}_{n}+\,\cos \,\theta \,{e}^{i{\varphi }_{\uparrow }(n-\mathrm{1)}}\,{\tilde{U}}_{n-1}=z\,{\tilde{U}}_{n},\quad n=2,\ldots ,\,N-1.$$

The missing equations for *n* = 1 and *n* = *N* are again supplied by the appropriate choice of BC: e.g. periodic or free boundary.

A few comments are in order. Firstly we notice that the phase *φ* in the original mapping (3) is irrelevant and can be put to zero throughout (as already noted in^6,7^). Secondly, in what follows we shall assume that *N* is large enough so that the system enjoys the self-averaging property as described later in the text. Finally, system (6) represents a random non-Hermitian eigenvalue problem. Note however that as the result of the performed double iteration the tridiagonal matrix $\hat{H}$ in the l.h.s. of Eq. (6) depends on the eigenvalue *z* itself. If *z* is an eigenvalue of the initial problem (4) then the eigenspectrum of $\hat{H}=\hat{H}(z)$ will contain both *z* and *N* − 1 spurious eigenvalues *ξ*_*α*_, *α* = 1, …, *N* − 1. Therefore in principle one can study the spectrum and localization properties of $\hat{H}$ alone with an independent complex spectral parameter *ξ* and at the end of the calculations put *ξ* = *z* to obtain the results relevant for the initial problem (4). The eigenproblem defined by the l.h.s. of (6) has interesting connections with other popular non-Hermitian problems recently discussed in literature. In particular the properties of the non-Hermitian tridiagonal operators of the type (6) have been studied in literature both analytically^30,31^ and numerically^32,33^. In fact a purely off-diagonal version of (6) was first introduced by by Feinberg and Zee in^34^ and later studied numerically in^32^. Formally the non-Hermitian hamiltonian of Feinberg and Zee can be obtained from $\hat{H}$ of the l.h.s. of Eq. (6) by taking the limit *θ* → 0 and *z* → ∞. On the other hand a tight binding model with complex *diagonal* disorder has been recently studied in the context of Anderson localization in dissipative waveguide arrays^35^. Further interesting properties of the non-Hermitian operator defined by the l.h.s. of Eq. (6) are studied in the Supplementary Information. However for the purposes of the present study there is no reason to consider a separate independent spectral parameter so in the following we shall only use a single notation *z*.

A powerful tool for studying localization properties in both Hermitian and non-Hermitian settings is the LE^29,36,37^ which can be defined by:

7 $$\lambda (z)=\mathop{\mathrm{lim}}\limits_{N\to \infty }\frac{1}{N}\,\mathrm{log}\,|{\tilde{U}}_{N+1}|=\mathop{\mathrm{lim}}\limits_{N\to \infty }\frac{1}{N}\,\sum _{n=1}^{N}\,\mathrm{log}|{r}_{n}|$$

In the above equation ${r}_{n}=\exp (-i{\varphi }_{\uparrow }(n)){\tilde{U}}_{n+1}/{\tilde{U}}_{n}$ is the so-called Riccati variable and the values of $\tilde{U}$ are obtained by iterating the second order recursion given by Eq. (6). In the absence of disorder the LE vanishes if *z* belongs to the spectrum of $\hat{H}$ since all the eigenmodes of this operator are extended. In the presence of disorder the finite value of *λ*(*z*) inside the spectral band *z* = exp(−*iβ*) provides an inverse localization length *l*_*loc*_ as pointed by Thouless^37^ (see also^36^). An important property of the LE is that it is a self-averaging quantity and is independent of the particular relization of disorder in the thermodynamical limit^29,36^.

From Eq. (6) one obtains the following complex recursion for *r*_*n*_:

8 $${r}_{n+1}=\frac{1}{\cos \,\theta }[{z}^{-1}+z\,{e}^{-i{\chi }_{n}}-\frac{\cos \,\theta \,{e}^{-i{\chi }_{n}}}{{r}_{n}}],\quad {\chi }_{n}\equiv {\varphi }_{\uparrow }(n+1)+{\varphi }_{\downarrow }(n)$$

From the above equation one can see that the statistical properties of the Lyapunov exponent and hence the localization length depend only on the effective total phase *χ*_*n*_. In particular in the case of full disorder the statistics of *χ*_*n*_ (modulo 2*π*) are exactly the same as the statistics of *ϕ*_↑_ and *ϕ*_↓_ alone. This means that in this case it is sufficient that only one component of the amplitude experiences phase disorder and put *ϕ*_↑_(*n*) or *ϕ*_↓_(*n*) to zero - without affecting the results. The case of full disorder is analyzed in more detail in the next section.

### Full disorder

In this section we shall prove that in the case of full disorder (i.e. Φ_*max*_ = *π*) the Lyapunov exponent (and hence the localization length) does not depend on the value of the spectral parameter *z*, as long as the latter remains inside the spectrum |*z*| = 1 and provide an explicit formula for the former.

We begin by noticing that if ${\tilde{U}}_{n}$ is a solution of Eq. (6) corresponding to *z* = exp(−*iβ*) then $z\,{\tilde{U}}_{n}$ is the solution of the same system with *z* = 1 and shifted phase disorder *ϕ*_↑↓_(*n*) → *ϕ*_↑↓_(*n*) + *β*. But in the case of full phase disorder the statistics of the solution are invariant under such phase shifts which means that if the state *z* = 1 has a value of LE *λ*(1) then all other states on the unit circle must necessarily have the same value and hence the same localization length. At the end of this section we shall provide an alternative proof of this fact using the direct recursion (8).

Next we shall use the fact that due to the self-averaging property the Lyapunov exponent can be calculated as the stationary average over the probability distribution of the Riccati variable, *λ*(*z*) = 〈log|*r*_*N*_|〉 where it is assumed that the statistics of |*r*_*N*_| become independent of *N* as *N* → ∞^32^. Such statistics can be obtained by iterating Eq. (8) starting from some arbitrary initial condition *r*_0_ and collecting enough samples to ensure that a final stationary distribution has been reached. Moreover in view of the previous paragraph is is sufficient to iterate Eq. (8) for *z* = 1 since LE is expected to be *z*-independent. The latter was indeed verified by taking *N* = 10^7^ iterations and scanning the spectrum (a unit circle in *z*) for different values of the coupling angle *θ*. In all the cases it was found that the relative difference between the maximum and minimum values of the LE across 100 spectral points calculated via Eq. (7) does not exceed 1% which can be attributed to residual numerical error and insufficient statistics.

It is relatively simple to obtain an analytical lower bound for the Lyapunov exponent directly from recursion (8). Indeed, since the phase *χ*_*n*_ is statistically independent from *r*_*n*_ we can calculate the conditional average **E**[log|*r*_*n*+1_| |*r*_*n*_] over the phase (assuming fixed value *r*_*n*_ from the previous iteration). The details can be found in the Methods section and the final result reads:

9 $${\bf{E}}[{\rm{l}}{\rm{o}}{\rm{g}}|{r}_{n+1}|{r}_{n}]=H[|1-\frac{\cos \,\theta }{{r}_{n}}|-1]\,{\rm{l}}{\rm{o}}{\rm{g}}|1-\frac{\cos \,\theta }{{r}_{n}}|-\,{\rm{l}}{\rm{o}}{\rm{g}}\,\cos \,\theta $$

where *H*[*x*] is the Heaviside function. Because of the Heaviside function the first term in the r.h.s. of the above expression is always non-negative which yields a uniform lower bound for the Lyapunov exponent: *λ*(*z*) ≥ |log cos *θ*|. This bound remains finite as long as *θ* > 0 which confirms that in the thermodynamic limit all eigenstates of the unit step operator ${\mathscr{U}}$ are exponentially localized in the regime of full disorder which is confirmed by the results of numerical simulations in Figs 1 and 2(a) and rigorous mathematical proofs in refs^22,23^.

**Figure 3:**
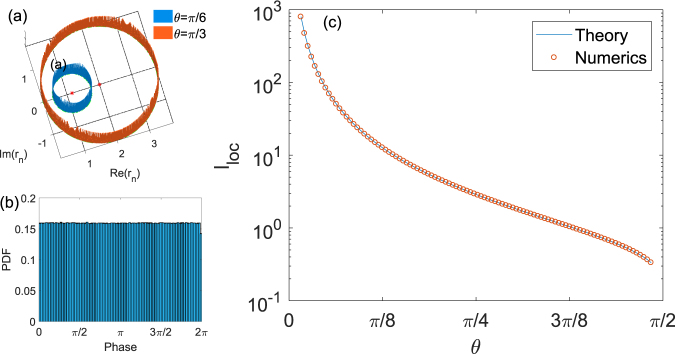
The histograms for the real and imaginary part of the Riccati variable (**a**) and phase of the reduced variable ***R***_*n*_ (**b**) for the case of full disorder. (**c**) Localization length as a function of the coupling angle *θ*.

We shall now prove that the above lower bound is not only tight but is in fact the exact value of the LE. To do so we first study the stationary statistics of the Riccati variable *r*_*n*_ obtained by iterating Eq. (8) with *z* = 1. We chose *N* = 10^7^ iterations and (in order to ensure that the memory from the initial condition has been lost) discard the first *N*/10 iterations. The resulting complex values of *r*_*n*_ have been binned as 2D histograms presented in Fig. 3(a) for two values of the coupling angle (other values have been tried as well producing similar results). One can immediately see that a limiting manifold of the map (8) has a peculiar structure of a circle with both the radius and position dependent on the angle *θ*. The position of the centre can be immediately inferred by taking the average of (8) with respect to the random phases *χ*_*n*_. The result is *R*_0_ = 1/cos *θ* which is marked in Fig. 3 by red star in the two cases. To understand the origin of the circular structure and find the expression for its radius let us rewrite the map (8) for *z* = 1 for the reduced variable *R*_*n*_ = *r*_*n*_ − *R*_0_. One has

$${R}_{n+1}={e}^{-i{\chi }_{n}}[\frac{{R}_{n}+{(\cos \theta )}^{-1}-\,\cos \,\theta }{\cos \,\theta \,{R}_{n}+1}]$$

The term in the brackets represents a linear fractional (Möbeus) transformation of the complex plane. It is known to transform circles to circles but more importantly it maps the circle |*R*_*n*_| = *R* = tan *θ* on itself. The random prefactor exp(−*iχ*_*n*_) does not break this symmetry and one can see that the limiting manifold of the Riccati map is indeed a circle ${\mathscr{C}}$ centered at *R*_0_ with the radius *R* perfectly matching the results of Fig. 3(a). The role of disorder in this situation is simply to randomize the phase of the reduced *R*_*n*_ variable and make the circle uniformly populated which is confirmed by the histograms presented in Fig. 3(b). The phase distributions for the reduced variable *R*_*n*_ for two different values of *θ* are almost indistinguishable and are very close to the uniform value *(2π*)^−1^ ≈ 0.16.

Returning to expression (9), it is easy to prove after simple algebra that the value of |1 − cos *θ*/*r*_*n*_| is equal to sin *θ* everywhere on ${\mathscr{C}}$ and therefore is always less than unity. This means that the first term in Eq. (9) vanishes as soon as the limiting manifold is reached and the Lyapunov exponent is given by the expression *λ*(*z*) = |log cos *θ*| which leads to the main result of this section:

10 $${l}_{loc}={\lambda }^{-1}(z)=1/|\mathrm{log}\,\cos \,\theta |.$$

The above result is in excellent agreement with that obtained numerically and shown in Fig. 3(c). The same result can also be obtained by averaging log|*r*_*n*_| directly over the limiting circle ${\mathscr{C}}$ assuming uniform distribution on this manifold. The resulting integral is of the type considered in the Methods section and one again arrives at the answer (10).

For the fair coin (*θ* = *π*/4, even power split) from (10) one has *l*_*loc*_ = 2/log 2 = 2.88 which is consistent with the exponential fall-off in Fig. 1(c). Eq. (10) also allows one to estimate the smallest possible coupling angle ${\theta }_{min}\ll 1$ for which the states are still localized for a system of size: ${\theta }_{min}\approx \sqrt{2}{N}^{-\mathrm{1/2}}$. Two final remarks are in order. Firstly it is possible to iterate map (8) for arbitrary values of the spectral parameter $z=\exp (-i\beta )$. The reduced variable is now $z\,{r}_{n}-1/\cos \,\theta$ and its mapping is exactly the same as for *z* = 1 (up to invariant rotation of the random phase). The limiting manifold is the same circle ${\mathscr{C}}$ only rotated around the origin by the angle *β*. Since the LE is determined by averaging the isotropic variable log|*r*_*n*_| such a rotation should have no effect on it which constitutes yet another proof that in the full disorder case the localization length is *z*-independent. Finally in the case of moderate or weak phase disorder when Φ_*max*_ < *π* one still expects a limiting manifold to be a circle ${\mathscr{C}}$. However in this case the isotropy is lost - the distribution of the points on the circle becomes non-uniform and the rotation w.r.t. to the origin which corresponds to different choice of spectral parameter *z* no longer leaves the disorder invariant which manifests in strong dependence *λ*(*z*). In the following section this dependence is studied perturbatively in the case of weak disorder ${{\rm{\Phi }}}_{max}\ll 1$.

### Weak noise expansion

Above we have demonstrated the main symmetries of the eigenvalue problem (4) and its equivalent (6) and the expression for the localization length in the case of full disorder. In this section we shall use small-noise pertrubation theory for the auxiliary tridiagonal Hamiltonian matrix *H* to obtain the spectral dependence of the LE.

The unperturbed hamiltonian corresponds to coherent DTQW with no noise (i.e. phase disorder) present (i.e. one puts all phase factors *ϕ*_↑,↓_ to zero in Eq. (6)). The perturbation is then given by the tridiagonal matrix ${\hat{H}}_{pert}=\hat{H}({\varphi }_{\uparrow ,\downarrow })-{\hat{H}}^{0}$. In the case of uniform distribution the variance of the phase is equal to $\langle {\varphi }^{2}\rangle ={{\rm{\Phi }}}_{max}^{2}/3$ and serves the genuine small parameter of our perturbative expansion. We notice that a generic non-vanishing matrix element of ${\hat{H}}_{pert}$ is proportional to *ε*_*n*_ = exp(*iϕ*_↑,↓_) − 1. To the first order in 〈*ϕ*^2^〉 it has only two non-vanishing moments 〈*ε*_*n*_〉 = −〈*ϕ*^2^〉/2 and $\langle {\varepsilon }_{n}^{2}\rangle =-\langle {\varphi }^{2}\rangle$ (notice the non-zero average). To obtain the perturbative expansion for the Riccati variable from Eq. (8) we use a standard method previously applied in both Hermitian^38^ and non-Hermitian^35^ settings. The details can be found in the Methods section and the final result reads:

11 $$\lambda (\beta )=\frac{\langle {\varphi }^{2}\rangle }{4}\frac{{\sin }^{2}\,\theta }{{\cos }^{2}\,\theta -{\cos }^{2}\,\beta }$$

**Figure 4:**
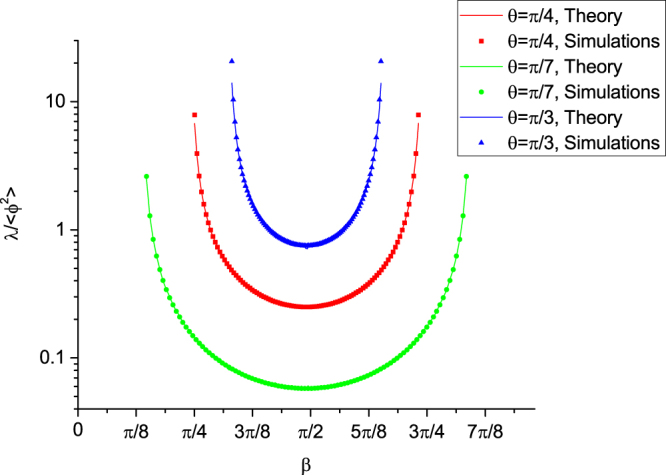
The normalized Lyapunov exponent given by Eq. (11) and the corresponding numerical results for three different values of the coupling angle.

Below in Fig. 4 we plot normalized Lyapunov exponent *λ*(*β*)/〈*ϕ*^2^〉 for Φ_*max*_ = 0.3 and several values of the coupling angle *θ*. The theoretical result is superimposed on the data from the numerical iteration of map (8) (*N* = 10^8^ steps were used) and appears to be in an excellent agreement agreement with the latter.

Therefore a good approximation of the minimum localization length achieved at the middle of the band *β* = *π*/2 is *λ*_*0*_ = (〈*ϕ*^2^〉/4)tan^2^*θ*. As seen from Fig. 4 it is quite close to the numerical value even in the presence of possible band anomaly (see the discussion at the end of the Methods section). One can clearly see the competition between the coupling and localization. In particular when the coupling angle tends to *π*/2 even weak localization effects can be enhanced and all the states in a *finite* system of size *N* will still appear strongly localized provided that the inequality $\langle {\varphi }^{2}\rangle \,{\tan }^{2}\,\theta \gg 1/N$ takes place.

### Absence of diffusion of the random quantum walker

**Figure 5:**
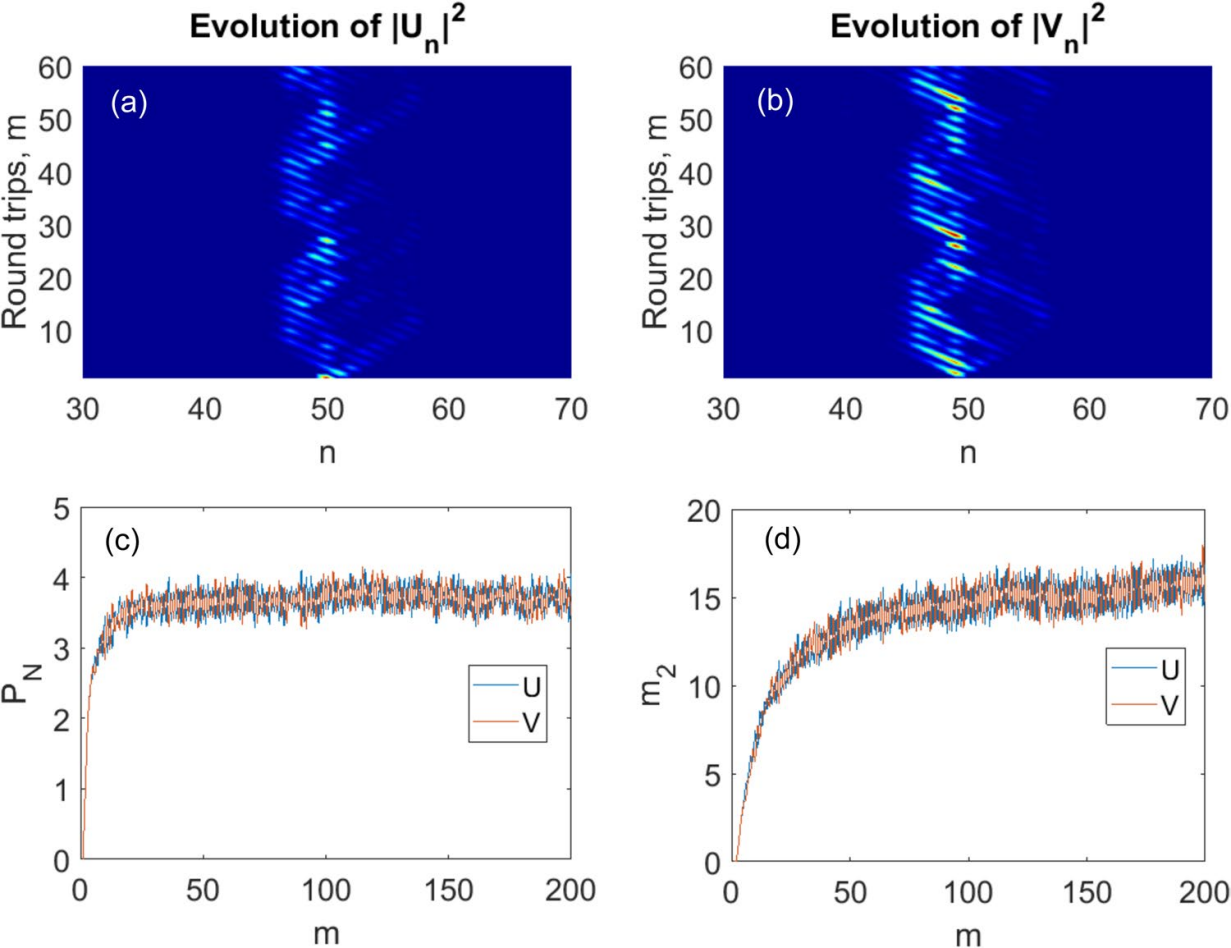
The spread of an initially localized amplitude (**a**,**b**) and the evolution of the averaged participation number (**c**) and the second moment (**d**) with the number of the round trips (quantum steps), *m*.

Some results on the subject of disorder-arrested diffusion have already been reported in^15^ with the rigorous mathematical background provided in refs^22,23^. Usually the main motivation for studying the stationary eigenproblem like (4) is to quantify the absence of diffusion in the dynamical propagation of an initially localized state. This corresponds to a quantum walker initially localized on a particular site or to a short classical pulse launched in one of the fibre loops at specific time mark *n*. Such an absence of diffusion was already observed numerically and experimentally in fibre-loop based DTQW with static phase disorder in ref.^15^. In this section we first repeat the simulations of ref.^15^ showing the absence of diffusion in the presence of disorder but then we also add the results for the simulations of the participation ratio dynamics as well as the simulations of the second moment of the solution defined as

12 $${m}_{2}(m)=\sum _{n=1}^{N}\,{(n-{n}_{0})}^{2}{|{\psi }_{n}^{m}|}^{2}$$

where *ψ* takes values *U* or *V* (upper or lower loop) and *n*_0_ is the first moment - i.e. the centre of the wave packet. Both participation ratio and the second moment provide quantitative measure for the lateral spread of the solution. The results are given in Fig. 5. We have assumed *N* = 100, *θ* = *π*/4, *φ* = *π*/2 and the system was initially excited in the state ${U}_{N\mathrm{/2}}^{0}=1$, *V*^*0*^_*n*_ = 0 with full disorder Φ_*max*_ = *π*. Periodic boundary conditions were assumed and a total number *m* = 200 of round trips (discrete steps) were performed. The results for the participation number and the second moment were additionally averaged over 200 Monte Carlo realizations to provide more statistics.

## Discussion

In this paper we have studied the phenomenon of Anderson localization of a particle moving performing quantum walk in the presence of random (but unitary) static phase disorder or equivalently evolution of classic light trapped in two connected fibre loops of unequal length in the presence of random modulation. We have shown that strong coupling between the two components of the wave-function (or field amplitudes in the two loops) facilitates localization and obtained an exact expression for the localization length in the case of strong phase disorder. We have also found the spectral dependence of the reciprocal localization length in the case of weak disorder by means of perturbative expansion of the Lyapunov exponent of the effective non-Hermitian eigenproblem. In particular the minimal reciprocal localization length was found to be proportional to the tan^2^*θ* - the ratio of the power coupling coefficients between the two components (two arms of the loop coupler) which can be very large for strongly biased quantum coin - making localization effects prominent even for small values of phase disorder. In addition in our theoretical analysis we have also studied the limiting manifold of the auxiliary map for the Riccati variable *r*_*n*_ and showed how its specific geometric structure affects the value of the Lyapunov exponent.

## Methods

### The derivation of Eq. (9)

In this section we shall perform a conditional averaging of log|*r*_*n*+1_| using expression (8) for the fixed value of the complex Riccati variable *r*_*n*_ and assuming *z* = 1. Introducing the amplitude and the phase of a complex number 1 − cos *θ*/*r*_*n*_ ≡ *A* exp(*iϕ*) one can write log|*r*_*n*+1_| = log|exp(*iχ*_*n*_) + *A* exp(*iϕ*)| −  log cos *θ*. Our task is then to average this expression over the uniform phase distribution of *χ*_*n*_. Since *χ*_*n*_ is considered to be uniformly distributed over the interval [0, 2*π*) one can immediately see that the average is insensitive to the phase *ϕ*. It is only left to evaluate an integral depending on a single real parameter:

$$I(A)={\bf{E}}[\mathrm{log}|{e}^{i\chi }+A|]=\frac{1}{2\pi }{\int }_{0}^{2\pi }\mathrm{log}|{e}^{i\chi }+A|\,d\chi $$

This can be done in several ways. One possible solution is to differentiate the above expression with respect to parameter *A*. After a standard substitution *ξ* = exp(*iχ*) the integral becomes over a unit circle in the complex plane. The integrand has two simple poles at *ξ* = 0 and *ξ* = −*A*. When *A* > 1 the second pole does not contribute to the integral while in the opposite case the contributions of the two poles exactly cancel each other leading to the result: *I*′(*A*) = *H*[*A* − 1]/*A*. To integrate the above it is sufficient to supply the value of *I*(*A*) at the boundary point *A* = 1. The latter turns out to be a table integral:

$$I\mathrm{(1)}=\frac{1}{2\pi }{\int }_{0}^{2\pi }\mathrm{log}|{e}^{i\chi }+1|\,d\chi =\frac{1}{2}\,\mathrm{log}\,2+\frac{1}{4\pi }{\int }_{0}^{2\pi }\mathrm{log}|1+\,\cos \,\chi |\,d\chi =0$$

where the last integral can be found in [ref.^39^, (4.225)]. Using this boundary value, integrating the expression for *I*′(*A*) and returning to the original notations results in Eq. (9) of the main text.

### Perturbative expansion of the Lyapunov exponent

The following method for obtaining the Lyuapunov exponent expansion is similar to that presented for the Hermitian Anderson localization in ref.^38^. It was also more recently applied to the problem of AL in the non-Hermitian diagonal disorder in ref.^35^.

The perturbation term *ε*_*n*_ = exp(*iχ*_*n*_) − 1 can be conveniently presented as *ε*_*n*_ = *εV*_*n*_ − *ε*^2^/2 where $\varepsilon =\sqrt{\langle {\chi }_{n}^{2}\rangle }$ is the small parameter of the expansion and the random variable *V*_*n*_ has zero mean and $\langle {V}_{n}^{2}\rangle =-1$ (up to the terms *O*[*ε*^4^]). Next we shall write down the Riccati eq. (8) separating the perturbation explicitly:

$${r}_{n+1}=\frac{1}{\cos \,\theta }[{z}^{-1}+z-\frac{\cos \,\theta }{{r}_{n}}]+[\frac{z}{\cos \,\theta }-\frac{1}{{r}_{n}}]\,(-\frac{{\varepsilon }^{2}}{2}+\varepsilon {V}_{n})$$

and use the fact that for large *n* the quantity log|*r*_*n*_| becomes self-averaging so that one has

$$\lambda (z)=\frac{1}{N}\,\sum _{n=1}^{N}\,{\rm{l}}{\rm{o}}{\rm{g}}|{r}_{n}|={\rm{R}}{\rm{e}}\langle {\rm{l}}{\rm{o}}{\rm{g}}\,{r}_{n}\rangle $$

Following a similar procedure for the Hermitian case^38^ let us seek the perturbed solution of the Riccati equation in the form

13 $${r}_{n}=A\,\exp \,[\varepsilon \,{B}_{n}+{\varepsilon }^{2}\,{C}_{n}+\ldots ]$$

It is important to note that *r*_*n*_ and hence the all the coefficients *B*_*n*_, *C*_*n*_ depend on the values of *V*_*i*_ with *i* < *n* therefore any averaging with respect to *V*_*n*_ can be performed separately from the average of the coefficients. Substituting this ansatz to the Riccati equation and keeping only the terms up to ${\varepsilon }^{2}=\langle {\chi }_{n}^{2}\rangle$ one obtains:

$$\begin{array}{cc}{\varepsilon }^{0}: & A=x-\frac{1}{A},\,\quad x\equiv \frac{1}{\cos \,\theta }[{z}^{-1}+z]\\ {\varepsilon }^{1}: & A{B}_{n+1}=\frac{1}{A}{B}_{n}+[\frac{z}{\cos \,\theta }-\frac{1}{A}]\,{V}_{n}\\ {\varepsilon }^{2}: & A\,[{C}_{n+1}+\frac{1}{2}{B}_{n+1}^{2}]=-\frac{1}{A}\,[-{C}_{n}+\frac{1}{2}\,{B}_{n}^{2}]-\frac{1}{2}\,[\frac{z}{\cos \,\theta }-\frac{1}{A}]+\frac{{B}_{n}}{A}\,{V}_{n}\end{array}$$

In the zeroth order one obtains a constant solution of the quadratic equation which gives a Lyapunov exponent of the unperturbed system: *λ*^(0)^(*z*) = ln|*A*|. Since the product of the two solutions is always equal to unity we must pick the solution which has the absolute value greater than unity so that the Lyapunov exponent is non-negative (in accordance with the general bounds for random complex Jacobi matrices given in^30^). Here we are interested in the case where *z* belongs to the unperturbed spectrum so that *x* is a real number in the interval [−2, 2]. Then both roots lie on the unit circle so that the zeroth order LE vanishes inside the spectrum as all the eigenstates are extended. As we shall see below there is a certain ambiguity in the choice of the the root depending on whether one approaches the spectral line from above or from below. In general one has

$${A}_{\pm }=A(x\pm i\mathrm{0)}=\frac{x\pm i\sqrt{4-{x}^{2}}}{2}\mathrm{.}$$

Next from the first order terms it follows that in the limit *n* → ∞ when the stationary distribution is reached one has

$$\langle {B}_{n+1}\rangle =\langle {B}_{n}\rangle =\langle B\rangle =0,\quad \langle {B}_{n+1}^{2}\rangle =\langle {B}_{n}^{2}\rangle =-\frac{{A}^{2}}{{A}^{4}-1}\,{[\frac{z}{\cos \theta }-\frac{1}{A}]}^{2}$$

Then averaging the equation for the *ε*^2^ terms we obtain

$$\begin{array}{ccc}\langle {C}_{n+1}\rangle =\langle {C}_{n}\rangle =\langle C\rangle  & = & -\frac{{A}^{2}+1}{2({A}^{2}-1)}\langle {B}^{2}\rangle -\frac{A}{2({A}^{2}-1)}\,[\frac{z}{\cos \,\theta }-\frac{1}{A}]\\  & = & \frac{{A}^{2}}{2({A}^{2}-{1)}^{2}}\langle {B}^{2}\rangle -\frac{A}{2({A}^{2}-1)}\,[\frac{z}{\cos \,\theta }-\frac{1}{A}]\end{array}$$

Thus for the Lyapunov exponent defined as *λ*(*z*) = ln|*A*| + *ε* Re〈*B*〉 + *ε*^2^ Re〈*C*〉 one obtains two possible choices:

$$\frac{{\lambda }_{\pm }(z)}{\langle {\chi }_{n}^{2}\rangle }=-\frac{1}{2}\frac{1}{4-{x}^{2}}[\frac{{x}^{2}-2}{2}+\frac{1}{{\cos }^{2}\,\theta }{\rm{R}}{\rm{e}}\frac{1}{{z}^{2}}-\frac{x}{\cos \,\theta }{\rm{R}}{\rm{e}}\frac{1}{z}]\mp \frac{1}{4}$$

Let us assume two-sided phase disorder so that $\langle {\chi }_{n}^{2}\rangle =2\langle {\varphi }^{2}\rangle$. Substituting the value of interest *z* = exp(−*iβ*), with |cos *β*| < cos *θ*, the above simplifies to:

14 $${\lambda }_{+}(\beta )=\frac{\langle {\varphi }^{2}\rangle }{4}\frac{{\sin }^{2}\,\theta }{{\cos }^{2}\,\theta -{\cos }^{2}\,\beta },\quad {\lambda }_{-}(\beta )=\frac{\langle {\varphi }^{2}\rangle }{4}\,[3+\frac{{\sin }^{2}\,\beta }{{\cos }^{2}\,\theta -{\cos }^{2}\,\beta }]$$

To pick the correct solution we need to recall that in the limit of zero coupling *θ* → 0 all the states are extended regardless of the presence of disorder so that the Lyapunov exponent should vanish. From this one concludes then that the correct choice is *λ*(*β*) = *λ*_+_(*β*).

It is important to emphasize that the perturbative expansion (13) is known to be singular at the points of the unperturbed spectrum which leads to band anomalies whenever the quantity *A* − 1 is a root of unity^38,40^. This includes the middle of the band *β* = *π*/2, *x* = 0 and one can study such anomalies in a fashion similar to that of the above-cited references. However as the first approximation the above result seems to be quite close (which is confirmed by numerical simulations of Fig. 4) and therefore we shall leave this analysis for future study.

### Data Availability

The datasets generated during and/or analysed during the current study are available from the author on reasonable request.

## Electronic supplementary material

Supplementary Information
