# Supplementary material for: Anderson localization of a one-dimensional quantum walker
Source: Sci Rep. 2018 Jan 29;8:1795. doi: 10.1038/s41598-017-18498-1 (PMC5788940; doi:10.1038/s41598-017-18498-1)
Supplement: Supplementary file 1 — Supplementary Information [file 41598_2017_18498_MOESM1_ESM.pdf]

Supplementary Information for “Anderson localization of a one-dimensional quantum walker”

Stanislav Derevyanko

THE 2D DENSITY OF STATES OF THE AUXILIARY TRIDIAGONAL NON-HERMITIAN MATRIX

In this Supplementary Information we study the structure of the 2D density of states (DOS) of the non-Hermitian tridiagonal matrix in Eq.(6) of the main text and how it affects the localization properties of the DTQW problem. Here for simplicity we shall restrict ourselves to the case of full disorder. It is a well-known fact that the DOS and the Lyapunov exponent are related by the so-called Thouless formula [1–3]. Looking specifically at the non Hermitian operator $\hat{H}$ in the l.h.s. of Eq.(6) and assuming it is diagonalizable with the set of eigenvalues ξ_α (one of which must coincide with z if the latter is in the spectrum of the original DTQW eigenproblem (4)) this result reads [2, 3]:

$$\lambda(\xi) = \int\int d\xi_1'\, d\xi_2'\, \rho(\xi_1', \xi_2') \log|\xi - \xi'| - \log\cos\theta \tag{1}$$

with the complex variable $\xi' = \xi_1' + i\xi_2'$ and the integration is extended over the support of the 2D DOS defined as $\rho(\xi_1, \xi_2) = N^{-1}\sum_\alpha \delta^{(2)}(\xi - \xi_\alpha)$. This formula can be proven in many ways e.g. using a standard co-factor expansion of the Green function [1, 4]. It was soon noticed that Eq.(1) is fully analogous to 2D electrostatics since it has the form of the solution of the 2D Poisson equation driven by a distribution of sources given by the 2D DOS $\rho(\xi')$. Therefore if one studies the structure and support of the DOS one can also gain some insight into the localization properties of the corresponding eigenstates. Of course for the QW the most relevant area is the vicinity of the the states with $\xi = z$ i.e. a unit circle but other models closely related to Eq(6) can also present an independent interest.

As mentioned in the main text one interesting model closely related to system (6) was provided by Feinberg and Zee [5] and corresponds to taking the limit $z \to \infty$ yielding a purely off-diagonal correlated gauge disorder (phase potential). If additionally one assumes purely imaginary phases (imaginary vector potential) this model transforms into the celebrated Hatano-Nelson model [6] (albeit with random coupling).

With this in mind let us consider the structure of the 2D density of states of the non Hermitian operator H in the l.h.s. of Eq(6) in the case of full disorder. From the symmetry of this operator it follows that if ξ_α is an eigenvalue of H then $\xi\,\exp(i\gamma)$ with real γ is an eigenvalue of the same operator but with the shifted angles: $\phi_{\uparrow\downarrow}(n) \to \phi_{\uparrow\downarrow}(n) + \gamma$, $z \to z\exp(i\,\gamma)$. But constant shift of the phases does not change their statistical properties

so for the density of states we obtain: $\rho(\xi; z) = \rho(\xi \exp(i\gamma); z \exp(i\gamma))$. Moreover it follows from the rotational symmetry above and expression (1) that the localization parameter is isotropic: $\lambda(z) = \lambda(|z|)$. Therefore without loss of generality we can study the spectrum of the operator $\hat{H}$ for $z = 1$.

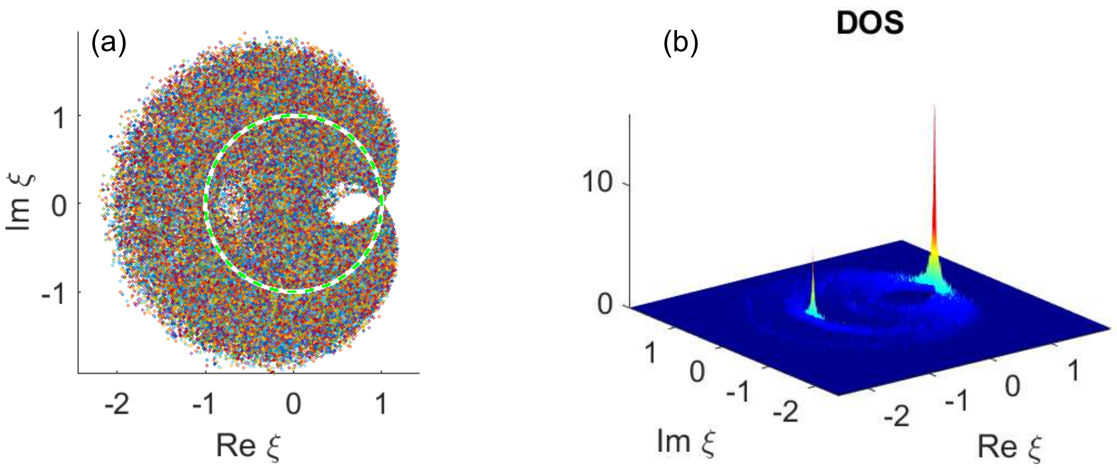

FIG. 1. The support of the eigenvalues (a) and the averaged 2D DOS (b) of system (6) of the main text with full disorder. The system size was $N = 100$, $\theta = \pi/4$ and 1000 random realizations of phases were used.

In Fig.1 we plot the numerically obtained support of the spectrum and the corresponding DOS. The unit circle is shown for reference. One can see that the support of the eigenspectrum of matrix $\hat{H}$ has an interesting "cardioid" shape with a symmetric gap to the left of the point $\xi = z = 1$. The averages DOS on the other hand shows a "tiara" shape with two pronounced peaks at $\xi = \pm 1$. It is interesting to compare the results presented in Fig.1 with those for the purely diagonal gauge disorder of Feinberg and Zee [5]. There it was established numerically [2, 5] that DOS is roughly unifrom on circle of radius $\approx \pi/2$ which is also close to the outer rim of the support of DOS in Fig.1. However the brief look at the structure of the DOS of the resulting tridiagonal problem reveals that DOS in this case is far from uniform. Using the electrostatic analogy of Eq.(1) one can see that the gap in DOS clearly visible in Fig.1 serves as a "lacuna" or otherwise a "negatively charged" area inside the full shape of the DOS support. Moreover the peaks and the outer rim of the "tiara" clearly contain most of the density which has an impact on the resulting LE. At present both the observed gap and the two peaks at $\xi = \pm z$ await full theoretical explanation which we leave for future study. Also there exist a version of the Thouless formula [7] that is valid

directly for the arbitrary disordered DTQW including the one considered in the main version of the text. Note that this formula differs from (1) since the latter describes the spectrum of the auxiliary operator (6) which among other properties does not possess the unitary symmetry of the original QW. The application of the Thouless formula for the original QW to calculating the LE $\lambda(z)$ is another interesting topic for future study.

---

[1] D.J. Thouless, in *Ill-condensed Maller*, (eds. Toulouse, G. & Balian. R.) 1–62 (Amsterdam: North-Holland, 1979).

[2] B. Derrida, J.L. Jacobsen, & R. Zeitak, J. Stat. Phys. **98**, 31 (2000).

[3] I.Ya. Goldsheid & B.A. Khoruzhenko, Isr. J. Math. **148**, 331 (2005).

[4] B. Kramer & A. MacKinnon, Rep. Prog. Phys. **56**, 1469 (1993).

[5] J. Feinberg & A. Zee, Phys. Rev. E **59**, 6433 (1999).

[6] N. Hatano & D.R. Nelson, Phys. Rev. Lett. **77**, 570 (1997).

[7] A. Ahlbrecht, V.B. Scholz & A.H. Werner, J. Math. Phys. **52**, 102201 (2011).
